# Feasibility of a Web-Based and Mobile-Supported Follow-Up Treatment Pathway for Adult Patients With Orthopedic Trauma in the Netherlands: Concurrent Mixed Methods Study

**DOI:** 10.2196/57579

**Published:** 2024-11-26

**Authors:** Gijs J A Willinge, Jelle F Spierings, Kim A G J Romijnders, Elke G E Mathijssen, Bas A Twigt, J Carel Goslings, Ruben N van Veen

**Affiliations:** 1 Department of Trauma Surgery, OLVG, Jan tooropstraat 164, Amsterdam, 1064 AE, the Netherlands, 31 615489516; 2 Department of Trauma Surgery, St. Antonius Ziekenhuis, Utrecht, the Netherlands; 3 Julius Center for Health Sciences and Primary Care, University Medical Center Utrecht, Utrecht University, Utrecht, the Netherlands; 4 The Healthcare Innovation Center, Julius Center for Health Sciences and Primary Care, University Medical Center Utrecht, Utrecht, the Netherlands

**Keywords:** musculoskeletal extremity injury, patient portal, follow-up treatment, healthcare utilization, patient experience, feasibility, orthopedics, trauma, Netherlands, mixed methods, resource utilization, electronic patient records, thematic analysis, qualitative data, digital treatment, mobile phone

## Abstract

**Background:**

Orthopedic trauma care encounters challenges in follow-up treatment due to limited patient information provision, treatment variation, and the chaotic settings in which it is provided. Additionally, pressure on health care resources is rising worldwide. In response, digital follow-up treatment pathways were implemented for patients with orthopedic trauma, aiming to optimize health care resource use and enhance patient experiences.

**Objective:**

We aim to assess digital follow-up treatment pathway feasibility from the patient’s perspective and its impact on health care resource use.

**Methods:**

A concurrent mixed methods study was conducted parallel to implementation of digital follow-up treatment pathways in an urban level-2 trauma center. Inclusion criteria were (1) minimum age of 18 years, (2) an active web-based patient portal account, (3) ability to read and write in Dutch, and (4) no cognitive or preexisting motor impairment. Data were collected via electronic patient records, and surveys at three time points: day 1‐3, 4‐6 weeks, and 10‐12 weeks after an initial emergency department visit. Semistructured interviews were performed at 10‐12 weeks post injury. Anonymous data from a pre-existing database were used to compare health care resource use between the digital treatment pathways and traditional treatment. Quantitative data were reported descriptively. A thematic analysis was used for qualitative data. All outcomes were categorized according to the Bowen feasibility parameters: acceptability, demand, implementation, integration, and limited efficacy.

**Results:**

Sixty-six patients were included for quantitative data collection. Survey response rates were 100% (66/66) at day 1‐3, 92% (61/66) at 4‐6 weeks, and 79% (52/66) at 10‐12 weeks. For qualitative data collection, 15 semistructured interviews were performed. Patients reported median satisfaction scores of 7 (IQR 6‐8) with digital treatment pathways and 8 (IQR 7‐9) for overall treatment, reflecting positive experiences regarding functionality, actual and intended use, and treatment safety. Digital treatment pathways reduced secondary health care use, with fewer follow-up appointments by phone (median 0, IQR 0‐0) versus the control group (median 1, IQR 0‐1; *P*<.001). Consequently, fewer physicians were involved in follow-up treatment for the intervention group (median 2, IQR 1‐2) than for the control group (median 2, IQR 1‐3; *P*<.001). Fewer radiographs were performed for the intervention group (median 1, IQR 0-1) than for the control group (*P*=.01). Qualitative data highlighted positive experiences with functionalities, intended use, and safety, but also identified areas for improvement, including managing patient expectations, platform usability, and protocol adherence.

**Conclusions:**

Use of digital follow-up treatment pathways is feasible, yielding satisfactory patient experiences and reducing health care resource use. Recommendations for improvement include early stakeholder involvement, integration of specialized digital tools within electronic health record systems, and hands-on training for health care professionals. These insights can guide clinicians and policy makers in effectively integrating similar tools into clinical practice.

## Introduction

Orthopedic trauma care faces significant challenges when it comes to adequate follow-up treatment and optimization of patient journeys [1,2]. These challenges include the often hectic situation surrounding patients’ initial visit in the emergency department (ED), the restricted provision and recall of medical information by patients, and variety in follow-up treatment pathways [3,4]. These challenges may impede patients’ understanding of their injuries and supplementary treatments, leading to inaccurate expectations and potentially hindering the recovery process and perceived engagement during follow-up treatment [3,5].

To address these challenges, the use of digital tools to support and empower patients during their patient journeys has significantly increased in recent years [2,6-10]. In the context of orthopedics, this has led to improvements of patient information provision, patient engagement, and overall patient journeys [2,6,11,12]. However, these tools have mainly been applied in chronic orthopedic care [9,13]. Their application in acute orthopedic trauma care remains limited. Moreover, existing digital tools lack interactive features that allow patients to actively shape their treatment, which could enhance care efficiency [14]. Therefore, we developed digital follow-up treatment pathways for patients with orthopedic trauma, comprising timed patient information, injury-specific patient-reported outcome measures (PROMs), and a specific questionnaire through which patients could directly influence scheduling of follow-up appointments. These pathways aimed to assist patients during their recovery, collect real time patient data, and optimize health care resource use by tailoring treatment to patients’ needs.

As this is a novel approach to trauma follow-up care in the Netherlands, evaluation of feasibility is crucial for sustainable development and implementation, especially considering the patient’s perspective as an end user [15]. Therefore, this study aimed to assess digital treatment pathways feasibility from a patient’s perspective and its effect on health care resource use. We hypothesized that the digital treatment pathways would be considered feasible by patients, while simultaneously reducing secondary health care use.

## Methods

### Design and Setting

A monocentric, explorative, prospective concurrent mixed method study was performed to assess the feasibility of newly implemented digital treatment pathways for patients with orthopedic trauma in an urban, level-2 trauma center and teaching hospital. A concurrent mixed methods triangulation design was used, using quantitative and qualitative research methods simultaneously with equal weight [16]. One team (GJAW and JFS) collected and analyzed quantitative data, and another team (KAGJR and EGEM) separately collected and analyzed qualitative data. Using the Bowen feasibility framework, both data types were categorized within the following themes: acceptability, demand, implementation, integration, and limited efficacy [17].

Our study was conducted among adult patients who received follow-up treatment through a digital treatment pathway at our institution between October 1, 2022, and April 1, 2023. Patients were eligible for a digital treatment pathway if they (1) were aged 18 years or older, (2) had an active account for the web-based patient portal, (3) were able to read and write in Dutch, and (4) had no cognitive impairment or preexisting motor impairment. Exclusion criteria were: multiple injuries, pathological fracture, and initial presentation or follow-up treatment at another institution. Additionally, a control group was formed using previously collected anonymous data from 100 consecutive patients to compare secondary health care use–related pre- and postimplementation outcomes of the digital treatment pathways. These data were collected between October 1, 2021, and February 10, 2022, as part of a clinical audit.

This study was reported according to the GRAMMS (Good Reporting on a Mixed Methods Study) criteria (Multimedia Appendix 1) and COREQ (Consolidated Criteria for Reporting Qualitative Studies; Multimedia Appendix 2) [18,19].

### Ethical Considerations

This study was ethically reviewed and approved by the Medical Ethical Committee of Utrecht, the Netherlands (WO 22.049). All included participants provided informed consent before study participation and all participants had the ability to opt out at any given time during the study. Prospectively collected data were deidentified and all retrospective data were used anonymously. Furthermore, all study data were stored on a secure institutional drive, only accessible by 2 researchers (GJAW and KAGJR). Study participants did not receive any compensation for their participation in this study.

### Treatment Specifics

Patients with orthopedic trauma at our institution are treated according to a virtual fracture care review protocol [20,21]. To optimize this protocol, additional digital treatment pathways were introduced for the 8 most frequent types of hand, wrist, ankle, and foot fractures on October 1, 2022, including both nonoperative and operative treatment (Table 1). Multimedia Appendix 3 offers a detailed description of the virtual fracture care review workflow and the digital treatment pathway.

**Figure 1.**
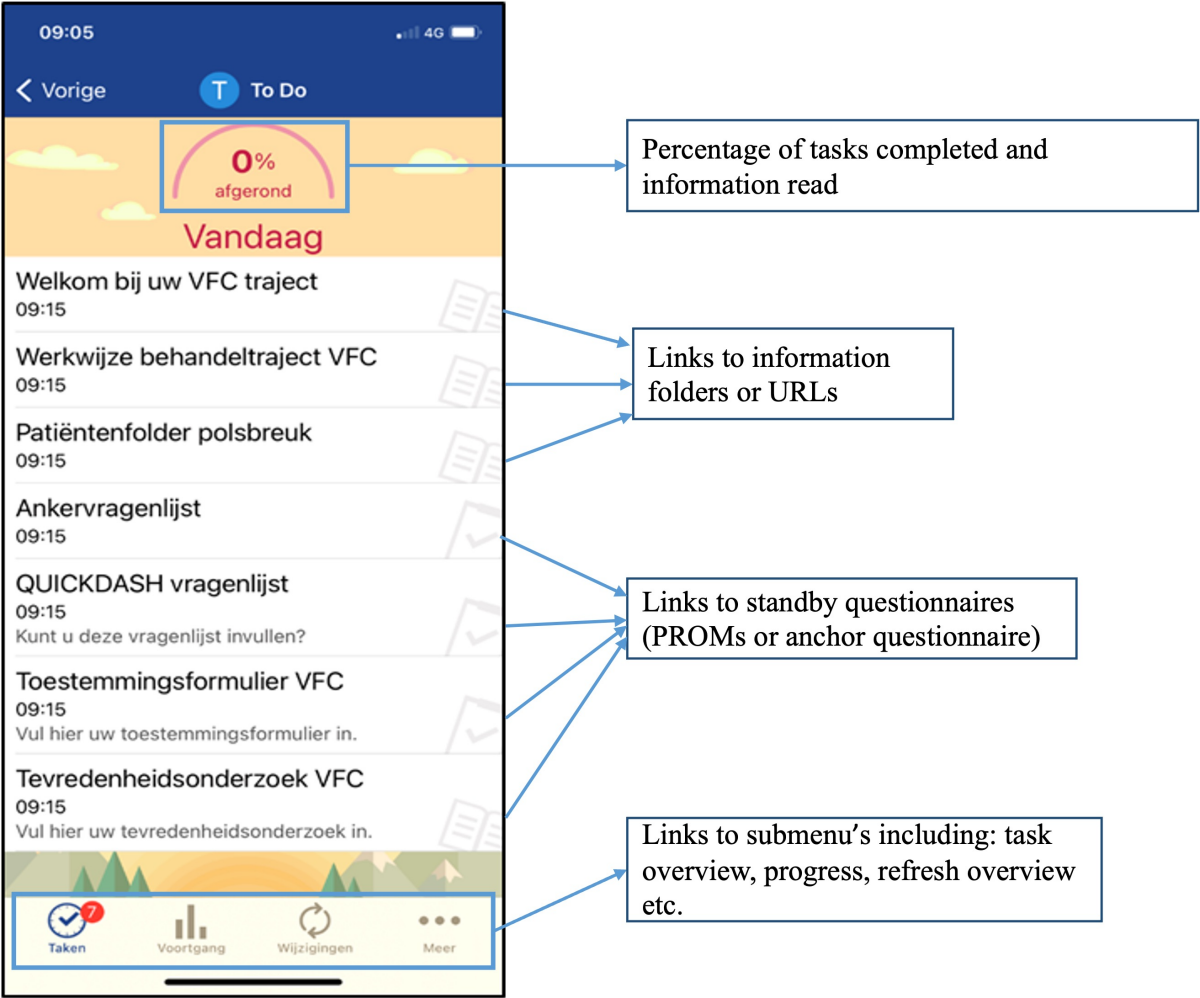
An impression of the layout of the menu within the patient portal and the accessory app. QUICKDASH: Quick Disabilities of the Arm, Shoulder, or Hand; PROM: Patient-Reported Outcome Measure; VFC: virtual fracture care.

The mode of delivery of the digital pathways was the web-based patient portal and its accessory app: “MyChart,” an extension of the electronic patient record (EPR) system: Epic (Epic Systems Corporation). An impression of its layout is provided in Figure 1.

**Figure 2.**
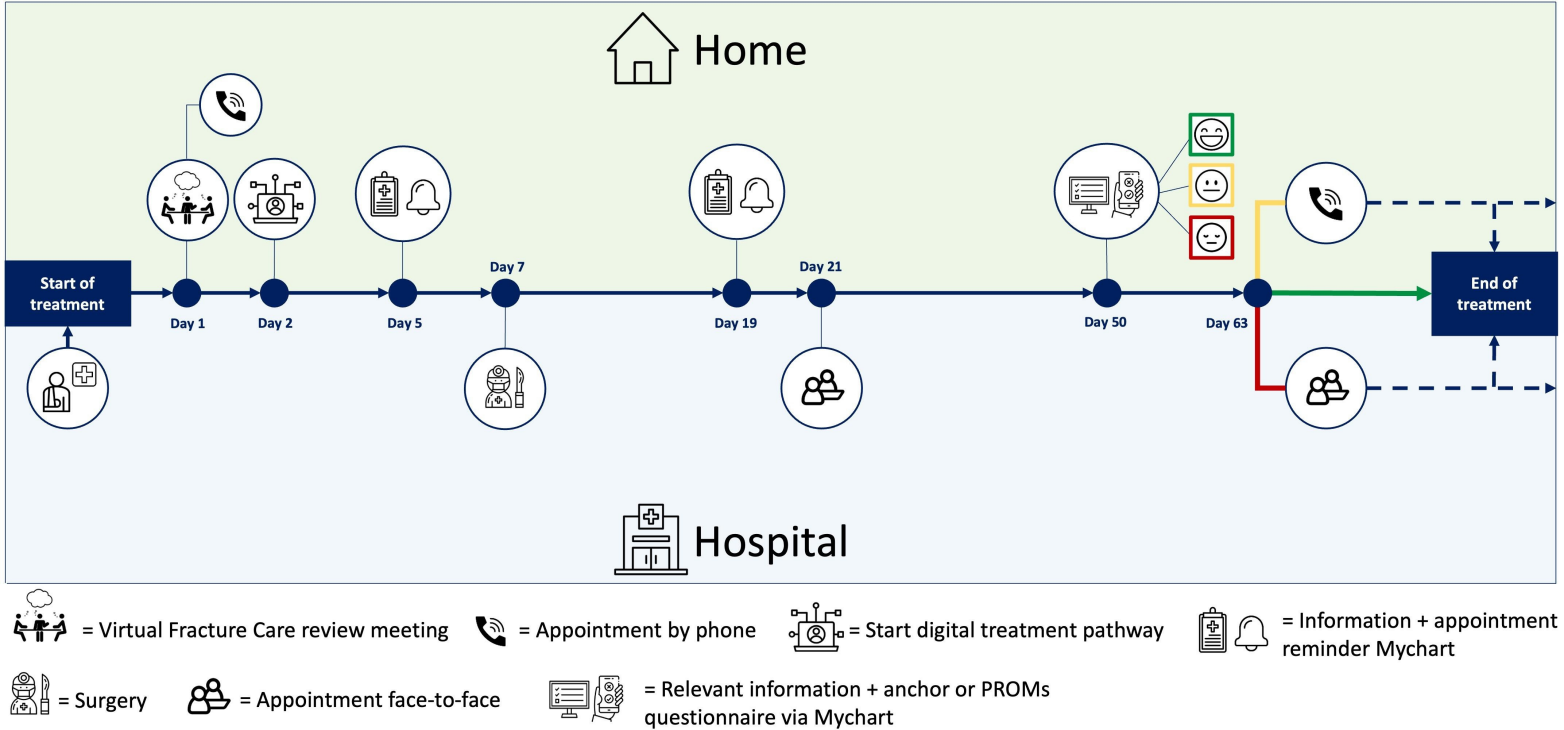
An example of a digital treatment pathway for an operatively treated distal radius fracture. PROM: Patient-Reported Outcome Measure.

In selected pathways, patients could directly influence their follow-up treatment through an additional anchor questionnaire sent prior to the final routine function check appointment. Based on the response, the need for an appointment was evaluated and planned accordingly, as shown in Figure S1 in Multimedia Appendix 3. Only patients who indicated stagnation or deterioration of recovery or dissatisfaction with their treatment received a follow-up appointment. Function check appointments that included imaging could not be guided by an anchor questionnaire, as this could not account for imaging results in its current form. An example of the course of a digital treatment pathway is provided in Figure 2.

**Table 1.** Injuries and treatment methods included in digital treatment pathways and used questionnaires.

Injury	Treatment	PROMs^a^	Timing (weeks)	Anchor questionnaire
Triquetrum fracture	Nonoperative	PRWE^b^	6-12-24	Yes
Scaphoid fracture	Nonoperative	PRWE	6-12-24	No^c^
Distal radius fracture	Nonoperative	QDASH^d^	6-12-24	Yes
Distal radius fracture	Operative	QDASH	7-13-24	Yes
Unimalleolar ankle fracture	Nonoperative	AOFAS^e^	6-12-24	No^c^
Unimalleolar ankle fracture	Operative	AOFAS	8-14-24	Yes
Bi or trimalleolar ankle fracture	Operative	AOFAS	8-14-24	Yes
Metatarsal fracture	Nonoperative	AOFAS	6-12-24	No^c^

a PROM: Patient-Reported Outcome Measure.

b PRWE: Patient Related Wrist Evaluation.

c These pathways did not include an anchor questionnaire-based function check appointment, as these function check appointments also included imaging and could therefore not be replaced in the digital treatment pathway.

d QDASH: Quick Disabilities of the Arm, Shoulder, or Hand.

e AOFAS: American Orthopedic Foot and Ankle Score.

### Sampling and Recruitment

The sample size for quantitative data collection was set at 70, based on general recommendations for the design of feasibility studies [17,22]. For the quantitative part, patients were recruited via convenience sampling. A researcher informed them about our study the day after their ED visit via an information email and by phone, and sent an information letter and informed consent form by email to patients indicating willingness to participate (Multimedia Appendix 4). Study participants provided digital informed consent before participation. Upon providing consent, patients also indicated willingness to partake in an interview. For the qualitative part of this study, patients were included using a purposive maximum variation sampling method to ensure heterogeneity in terms of gender, age, type of injury, and treatment strategy. The qualitative sample size was guided by the principle of data saturation and determined a posteriori [23]. The expected number of interviews was 15 [23].

### Data Collection

#### Overview

Data were collected from system data (EPR), surveys, and semistructured patient interviews.

#### System Data

Quantitative system data were collected 6 months after treatment initiation, including age, sex, smoking status, fracture type and treatment, PROMs and anchor questionnaire results, protocol compliance, follow-up appointments (face-to-face or by phone, with each contact counting as one appointment), health care professionals involved in follow-up treatment (physician or casting technician), follow-up imaging (radiograph, computed tomography scan, and magnetic resonance imaging scan), and ED reattendances and registered complications. The control group data were similar.

#### Surveys

Three web-based surveys were used to prospectively gather quantitative data, comprising a total of 38 questions. Surveys included 4-point Likert Scales (1=totally disagree to 4=totally agree), Visual Analogue Scales (0=extremely dissatisfied to 10=extremely satisfied), and free-text questions. The surveys were developed by 3 researchers (JFS, GJAW, and EGEM) and checked by 3 experts: a (orthopedic) trauma professor (JCG), a (orthopedic) trauma surgeon (RNvV), and an associate professor in process evaluations of health care innovations (JCA Trappenburg, PhD). Patients received surveys at three time points; day 1‐3 (T0), 4‐6 weeks (T1), and 10‐12 weeks (T2) after the ED visit (Multimedia Appendix 5). Patients who did not complete questionnaires received up to two reminder emails, sent after 3 and 5 days. All respondents per time point were included in the final analysis.

#### Semistructured Interviews

Qualitative data were collected through semistructured interviews using Microsoft Teams. The research team developed an interview topic list guide in collaboration with a variety of health care professionals (ED caregivers, casting technician, orthopedic trauma surgeon, and surgical resident) to explore patients’ perspectives (Multimedia Appendix 6). Interviews lasted approximately 45 minutes and were conducted by KAGJR an academic social scientist or EGEM an academic nursing scientist, 3 months after injury (T2). Open-ended questions were used to stimulate participants’ own interpretation and prompts were used to encourage deliberation. Interviews were conducted in Dutch and were audio recorded.

### Data Analysis

#### Quantitative Analysis

Quantitative data were analyzed using IBM SPSS (version 27; IBM Corp). Data were reported using frequencies with percentages for categorical variables, and continuous variables as mean with SD or median with IQR, depending on data distribution. Statistical significance was assessed using a chi-square test for categorical variables and either a paired *t* test (2-tailed) or Mann-Whitney *U* test for continuous variables, based on data distribution.

#### Qualitative Analysis

After 5 interviews, posteriori data saturation was assessed. This was reached after completing 14 interviews. The 15th interview planned was still conducted. Audio recordings were transcribed verbatim. Data were systematically analyzed by KAGJR and EGEM following the steps of thematic analysis to reflect data transformation [24,25]. NVivo (version 20; Lumivero) was used for data analysis. The main analysis involved 6 phases to identify recurring patterns and perspectives. Relevant themes and subthemes were deduced from the first 5 interviews (step 1), forming the basis for a coding list, which was used to code an entire transcript independently (step 2). This was then used to check for additional relevant codes during a joint meeting (step 3). Differences in coding were resolved to establish a final coding taxonomy (step 4), which was used to code all transcripts (step 5). Dutch quotes were translated to English using the forward backward method, and were used to illustrate themes (step 6).

#### Triangulation

After separate analyses, quantitative and qualitative data were triangulated through a Pillar Integration Process to highlight distinctive themes within data and identify similarities and differences between data sources [26]. The triangulation session was attended by both research teams and two (orthopedic) trauma surgeons (RNvV and BAT). One researcher (GJAW) presented quantitative results per study parameter, while another (KAGJR) presented qualitative findings.

## Results

### Demographics

**Figure 3.**
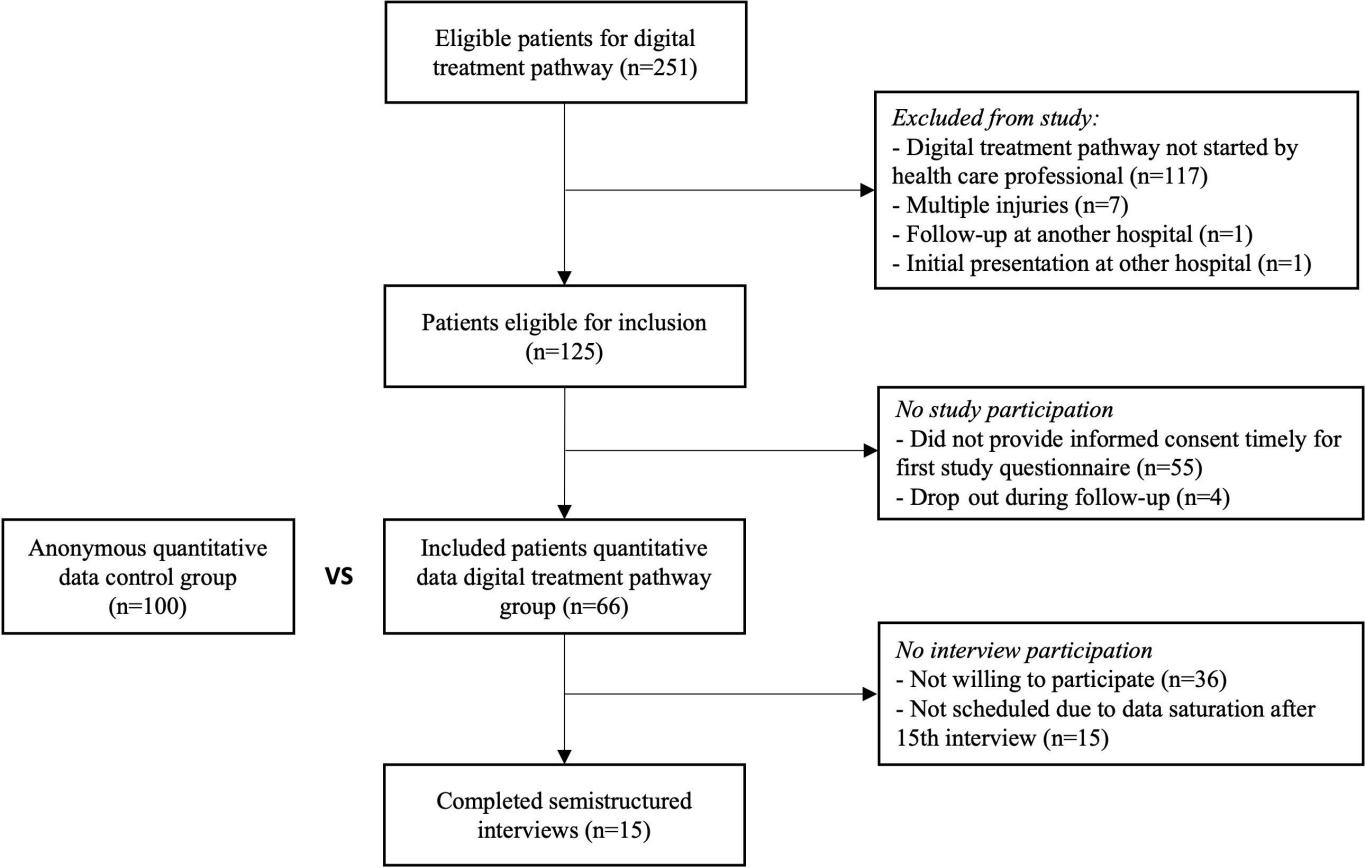
Flowchart of patient inclusion.

In total, 251 patients were eligible for a digital treatment pathway (Figure 3).

Of these patients, 125/251 (49.8%) were eligible for study inclusion, of whom 66/125 (52.8%) were included. Baseline characteristics are shown in Table 2.

Similar characteristics were compared between the control and digital treatment pathway groups, and no differences were identified. All included patients (66/66, 100%) completed the T0 study survey, 61/66 (92%) completed the T1 survey and 52/66 (79%) completed the T2 survey. The respondent groups at various time points did not differ in baseline characteristics (Table 2).

Thirty of 66 (45%) patients consented to interview participation of whom 15/66 (23%) were interviewed.

The reasons for not participating were not obtained. Nine out of 15 (60%) interviewees were female and 6/15 (40%) were male. Interviewee age ranged between 23 and 77 (median 48, IQR 35-62) years.

**Table 2.** Baseline characteristics of adult patients with orthopedic trauma included in a mixed method evaluation of digital treatment pathways for follow-up treatment of a musculoskeletal extremity injury.

Variables		T0^aa^ (*n*=66)	T1^b^ (*n*=61)	T2^c^ (*n*=52)	*P* value		
					T0-T1	T0-T2	T1-T2
**Sex, n (%)**					.93	.83	.89
	Male	26 (39)	23 (38)	19 (37)			
	Female	40 (61)	38 (62)	33 (63)			
Age (years), median (range)		53 (20-77)	53 (20-77)	57 (22-77)	.80	.09	.15
**Smoking, n (%)**					.98	.99	.99
	No	50 (75)	46 (75)	39 (75)			
	Former	0 (0)	0 (0)	0 (0)			
	Current	3 (5)	2 (3)	2 (4)			
	Unknown	13 (20)	13 (21)	11 (21)			
**Type of fracture and treatment, n (%)**					>.99	>.99	>.99
	Triquetrum conservative	5 (7)	5 (8)	4 (8)			
	Scaphoid conservative	0 (0)	0 (0)	0 (0)			
	Distal radius conservative	23 (35)	23 (38)	21 (39)			
	Distal radius operative	18 (27)	16 (26)	14 (27)			
	Metatarsal shaft conservative	3 (5)	2 (3)	2 (4)			
	Unimalleolar ankle conservative	5 (7)	4 (7)	2 (4)			
	Unimalleolar ankle operative	10 (15)	9 (15)	7 (14)			
	Bi or trimalleolar ankle operative	2 (3)	2 (3)	2 (4)			
**Treatment strategy, n (%)**					.82	.80	.97
	Nonoperative	36 (55)	35 (57)	30 (58)			
	Operative	30 (45)	26 (43)	22 (42)			

a T0: 1-3 days after emergency department visit.

b T1: 4-6 weeks after emergency department visit.

c T2: 10-12 weeks after emergency department visit.

### Acceptability

#### Overview

Acceptability was assessed through quantitative and qualitative evaluation of patients’ expectations of digital treatment pathways and its anticipated functionalities and advantages. Positive and negative patient experiences, along with influencing factors, were identified. Finally, patient satisfaction was evaluated.

#### Expectations

**Figure 4.**
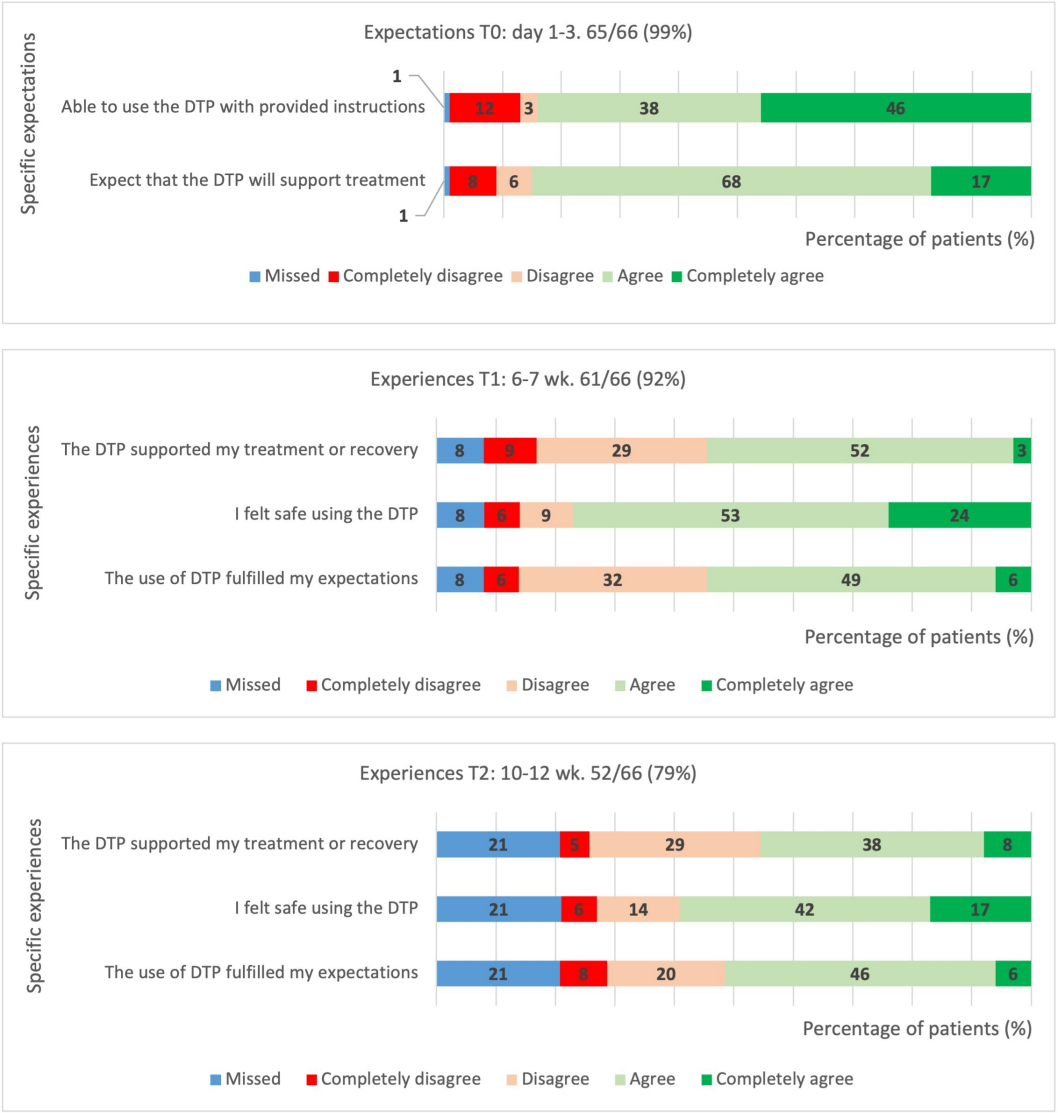
Likert scales acceptability: expectations and experiences. DTP: digital treatment pathway; T0: 1-3 days after emergency department visit; T1: 4-6 weeks after emergency department visit; T2: 10-12 weeks after emergency department visit.

Participants predominantly expected to be able to successfully use the digital treatment pathway (Figure 4). Additionally, 56/66 (85%) respondents expected that the digital treatment pathway would assist them throughout their treatment and 46/66 (70%) expected this would positively impact communication with their treatment team.

These expectations were further specified in qualitative data, including easy scheduling of follow-up appointments, insight into patient records (ie, summaries of visits and images results), and information on their fracture and recovery process. Some interviewees emphasized their initial expectations were not met, partly due to insufficient expectation management (Multimedia Appendix 7, quote 1). Additionally, participants acknowledged having lower expectations for this application compared to commercial apps, recognizing the unique context of a health care provider application.

#### Experiences

Survey respondents predominantly reported the digital treatment pathway had helped them during their treatment, with 36/61 (60%) at T1 and 30/52 (58%) at T2 (Figure 4). This was significantly lower compared to T0 with 56/66 (85%; *P*<.001). A total of 21/61 (35%) patients indicated their expectations were not met at T1, and 13/52 (25%) at T2. Respondents predominantly perceived the digital treatment pathways as safe, with 51/61 at T1 (83%) and 39/52 at T2 (75%).

These results were supported in qualitative data, as interviewees shared the functionalities of the app supported their follow-up treatment. The possibility to schedule and track appointments was particularly experienced as positive (Multimedia Appendix 7, quote 2). Negative experiences were linked to limited user-friendliness, difficult login and registration processes, and difficulty with finding desired information. Despite unmet expectations, overall experiences with received care were mainly positive (Multimedia Appendix 7, quote 3).

#### Satisfaction

Quantitative data showed a median satisfaction score with digital treatment pathways of 7 (IQR 6‐8) at T1, increasing to 7.5 (IQR 7‐9) at T2. A similar score was observed for satisfaction with overall treatment team communication, with a median of 7 (IQR 6‐8) at both T1 and T2. Additionally, overall treatment scores at T2 were higher than scores reported specifically for the digital treatment pathway, with a median score of 8 (IQR 7‐9) for overall treatment satisfaction and a median score of 8 (IQR 7‐8.5) for overall information provision.

During interviews, participants were generally satisfied with the digital treatment pathways. This was mainly based on the appointment scheduling and reminder or notification feature. One of the most frequent reasons provided for a relatively low score was the unclear layout and menu of the application, complementing quantitative data. Participants clearly indicated this would need to be improved to increase their satisfaction level (Multimedia Appendix 7, quote 4).

### Demand

#### Overview

Demand was both quantitatively and qualitatively assessed through patients’ perceived capability to use digital treatment pathways effectively and factors facilitating and hindering adequate use. Additionally, patients’ intended use of digital treatment pathways in the future was evaluated, along with their likelihood to recommend this to others.

#### Actual Use

**Figure 5.**
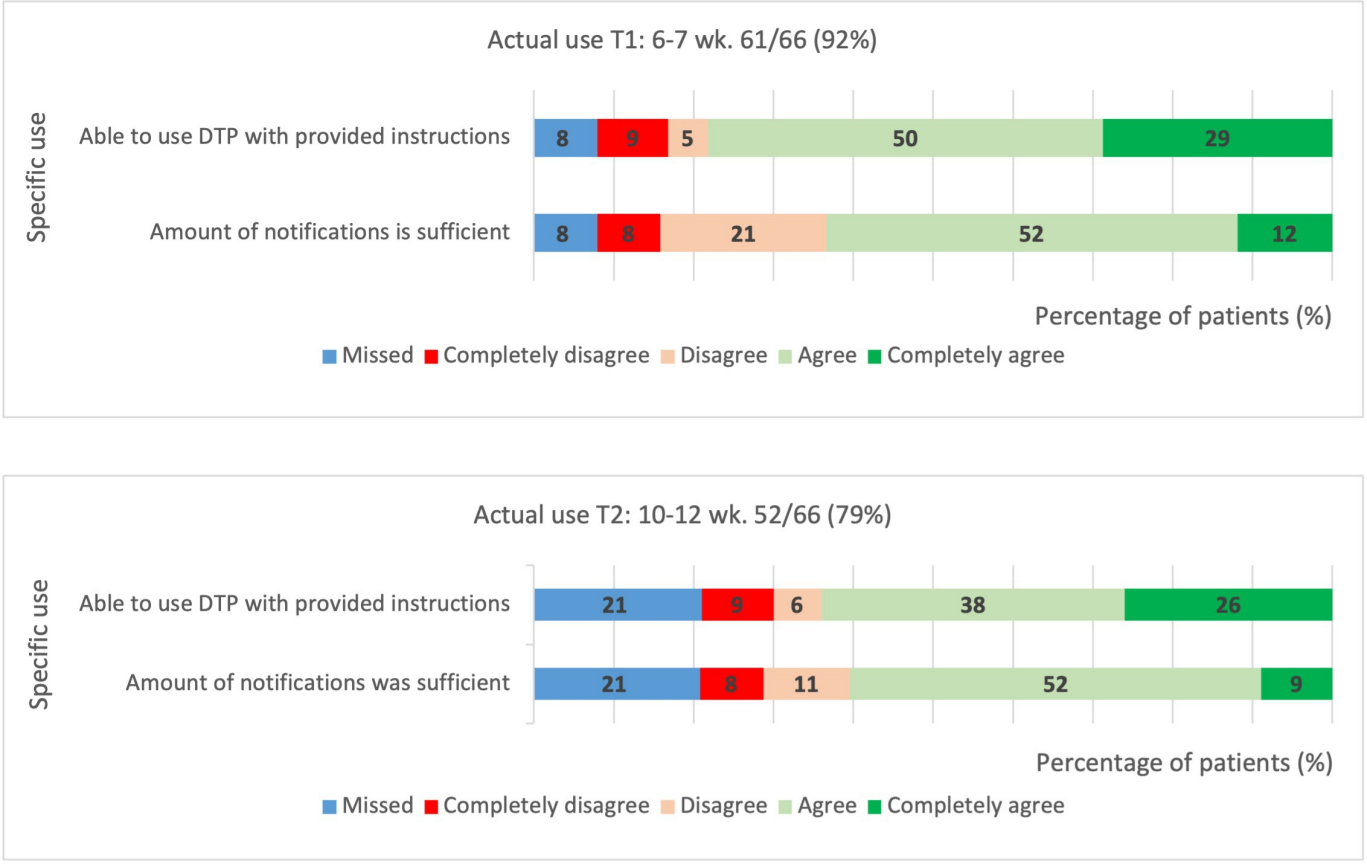
Likert scales demand: actual use. DTP: digital treatment pathway T1: 4-6 weeks after emergency department visit; T2: 10-12 weeks after emergency department visit.

During follow-up treatment, participants predominantly felt able to use the digital treatment pathway with provided instructions, with 52/61 (85%) positively reporting on this at T1 and 42/52 (81%) at T2 (Figure 5).

The majority of patients found received notifications sufficient throughout follow-up, with 39/61 (64%) at T1 and 32/52 (61%) at T2. Respondents who considered notifications insufficient expressed a need for more information and supportive notifications in the later stages of follow-up (eg information and exercise instructions after the first month of recovery).

During the interviews, participants shared they were able to use the MyChart app well, as its features were familiar to smartphone users. However, several barriers were also shared; the main barrier being the confusing layout of the application. This hindered patients in finding the right information or functions (Multimedia Appendix 7, quote 5).

#### Intended Future Use

Respondents generally expressed a high likelihood of using digital treatment pathways in the future, with a median score of 9 (IQR 7-10) at T1 and a median score of 8 (IQR 7-10) at T2. Regarding the likelihood of recommending the digital treatment pathways to others, patients reported a median score of 8 (IQR 6-9) at T1 and 8 (IQR 6-9) at T2.

Interviewees reported similarly positive outlooks. They were convinced that the future of health care would become more digital and regarded digital treatment pathways as a great starting point. From a practical view, interviewees shared they would use it again, if only to keep track of appointments (Multimedia Appendix 7, quote 6).

### Implementation

#### Overview

The success of the implementation was assessed by health care professionals’ compliance with the new protocol and patients’ completion of questionnaires in the digital treatment pathway. Factors perceived by patients as facilitating or hindering implementation were also identified, with results from both data sources included.

#### Protocol Compliance and Technical Issues

During inclusion, 117/251 (46.6%) eligible patients did not receive a digital treatment pathway due to lack of initiation by health care professionals. No technical issues were encountered.

#### PROM Completion

The completion rate of PROM questionnaires was substantial, although it exhibited a decrease as the follow-up duration extended (Table 3).

**Table 3.** Questionnaire completion among adult patients with orthopedic trauma and protocol compliance by health care professionals for follow-up treatment of a musculoskeletal extremity injury with a digital treatment pathway.

Outcomes		Digital treatment pathway (n=66), n (%)
**Completion of PROMs** ^**a**^		
	6‐8 wk completed	60 (91)
	12 wk completed	56 (85)
	24 wk completed	44 (67)
**Anchor questionnaires sent**		52 (79)
	Completed	48 (92)
	Missed	4 (8)
**Anchor questionnaire results**		
	No appointment	40 (77)
	Phone appointment^b^	11 (21)
	Face-to-face appointment	1 (2)
**Anchor questionnaire compliance by HP** ^**c**^		
	Compliant	33 (63)
	Noncompliant	19 (37)
**Type of protocol deviation**		
	Extra appointment	10 (19)
	Extra appointment and radiograph	9 (17)
**Reason for protocol deviation**		
	Unknown^d^	14 (27)
	Patient’s request	1 (2)
	Clinical indication	4 (8)

a PROM: Patient Reported Outcome Measure.

b Four phone appointments due to incomplete or missed anchor questionnaire.

c HP: health care professional.

d This was attributed to unawareness or noncompliance to protocol.

#### Anchor Questionnaire Compliance and Results

Of the 66 patients, 52 (79%) received an anchor questionnaire (Table 3). Eleven out of 66 (17%) patients were ineligible due to the type of injury and 3/66 (4%) were not willing to participate in the anchor questionnaire. These patients were planned for a routine function check follow-up appointment by phone. The 4/66 (8%) patients who did not complete the questionnaire were also planned for function check follow-up by phone. Following the questionnaire results, 40/52 (77%) of patients did not need a follow-up appointment. However, health care professionals deviated from the protocol in 19/52 (37%) of patients and planned a follow-up appointment despite the questionnaire results. For 14/19 (74%) of protocol deviations, no clear indication was documented. These were thus attributed to protocol unawareness by health care professionals.

#### Barriers and Facilitators to Implementation

Barriers shared by interviewees included limited user-friendliness of the patient portal and the MyChart app, the required digital skills, and the linguistic orientation of the platform. Facilitators included patients’ positive attitudes toward digital supportive tools and the flexibility for patients to answer questions, influence their treatment, and access information at their convenience (Multimedia Appendix 7, quote 7). Furthermore, understanding the efficiency it offered health care providers increased interviewees’ willingness to use the digital treatment pathways.

### Integration

This was assessed though qualitative data provided by interviewees focusing on the ease of integration of digital treatment pathways into patients’ daily activities. Interviewees generally found the MyChart app easy to incorporate into their lives, noting that its mode of delivery was familiar to smartphone users. However, they pointed out that successful integration depended on users’ digital proficiency. Furthermore, participants mentioned that the app’s language settings and layout made it time-consuming to navigate to the desired section, which could be challenging during daily activities such as work, hindering a seamless integration (Multimedia Appendix 7, quote 8).

### Limited Efficacy

Limited efficacy was assessed by comparing secondary health care resource use before and post implementation of the digital treatment pathways. This was complemented by patients’ perspectives on the necessity of function check appointments and the new scheduling process through the anchor questionnaire.

Quantitative data showed a reduction of follow-up appointments following the implementation of the digital treatment pathways (*P*<.001; Table 4).

**Table 4.** Secondary health care use and complications for adult patients with orthopedic trauma treated for a musculoskeletal extremity injury before and post implementation of a digital follow-up treatment pathway.

Variables		Control (n=100)		DTP^a^ (n=66)		*P* value
		n (%)	Median (IQR)	n (%)	Median (IQR)	
**Follow-up appointments**		429 (100)	4 (2-5)	192 (100)	3 (2-4)	<.001
	Phone appointments	128 (29.8)	1 (0‐1)	39 (20.3)	0 (0‐0)	<.001
	Face-to-face appointments	301 (70.2)	2 (1-4)	153 (79.7)	2 (1-4)	.07
**Involved caregivers**		429 (100)	4 (2-5)	192 (100)	3 (2-4)	<.001
	Physician	274 (63.9)	2 (1-3)	111 (57.8)	2 (1-2)	<.001
	Plaster technician	149 (34.7)	1 (0‐2)	79 (41)	1 (0‐1)	.21
	Physician assistant	6 (1)	0 (0‐0)	2 (1)	0 (0‐0)	.37
**Imaging**						
	Radiograph	128 (97)	1 (0‐1)	56 (93)	1 (0‐1)	.01
	CT^b^ scan	4 (3.0)	0 (0‐0)	3 (5)	0 (0‐0)	.87
	MRI^c^ scan	0 (0)	0 (0‐0)	1 (2)	0 (0‐0)	.22
ED^d^ reattendances		5 (5)	—^e^	1 (1)	—	.24
**Complications**						
	Persisting pain or stiffness reported^f^	19 (19)	—	8 (12)	—	.24
	Infection	0 (0)	—	1 (2)	—	.76

a DTP: digital treatment pathway.

b CT: computed tomography.

c MRI: magnetic resonance imaging.

d ED: emergency department.

e Not applicable.

f Based on reported persisting pain or stiffness at the last follow-up appointment within the 6 month follow-up period.

Primarily, this was the result of a reduction of appointments by phone. Control group patients had a median of 1 (IQR 0‐1) follow-up appointment by phone versus 0 (IQR 0‐0) in patients treated with a digital treatment pathway (*P*<.001). The number of face-to-face appointments remained similar with a median of 2 (IQR 1‐4) in the control group versus 2 (IQR 1‐4) in the digital treatment pathway group (*P*=.07). Fewer health care professionals, particularly physicians (*P*<.001), were involved in the follow-up of the intervention group compared to the control group. Patients in the digital treatment pathways had fewer follow-up radiographs (*P*=.01), while the number of computed tomography and magnetic resonance imaging scans remained unchanged. ED reattendances were rare in both groups, mostly due to cast-brace issues. No differences in complaints or complications were observed within 6 months. One patient in the intervention group developed a wound infection, treated with surgical debridement and antibiotics, leading to full recovery. This patient independently sought care, unaffected by the anchor questionnaire.

Qualitative data revealed varying outcomes regarding the need for follow-up appointments among participants. Some individuals found follow-up appointments unnecessary, while others deemed them essential (Multimedia Appendix 7, quote 9). Another participant shared an experience around persistent pain and mobility issues that were not picked up by the questionnaire (Multimedia Appendix 7, quote 10). This participant scheduled a follow-up appointment on their own initiative after receiving questionnaire feedback, emphasizing the importance of this option.

## Discussion

### Principal Findings

Our findings show that using a digital treatment pathway for patients with orthopedic trauma with extremity fractures is feasible, reflected in high usage, satisfaction, positive experiences, and reduced health care use. Participants saw it as a promising step toward digitizing trauma care, expressing willingness for future use. Nevertheless, clear areas for improvement were identified, including patient expectation management, user-friendliness of the patient portal and accompanying application, the implementation process, and protocol compliance by health care professionals.

### Patient Expectations and Experiences

The positive expectations and readiness among patients toward the digital treatment pathways, as reflected by the high questionnaire completion rates, aligns with other studies on the use of digital tools and home-monitoring within orthopedic patients [27-29]. The decline in completion rates during later stages of follow-up is a known challenge within this population, suggesting this it is not unique to the digital treatment pathways but rather associated with other characteristics of the orthopedic trauma population [30].

Although results were predominantly positive, approximately one-third of survey respondents expressed unmet expectations, a sentiment reinforced by interview findings. This was primarily due to a lack of user-friendliness, a common barrier in digital health care innovation [31]. In our study, this was attributed specifically to the web-based patient portal and the accessory MyChart app, both an extension of our EPR system. While integration with the EPR enables direct digital feedback, these systems were not designed for home monitoring, introducing usability issues [8,15,32-35]. Furthermore, these systems are generally used hospital-wide, requiring a wide array of different features and functions for different patient types. This could confuse patients, as confirmed by our participants. Specialized digital tools and pathways designed from an end user perspective could enhance usability and improve this mode of care [8,9]. Such digital tools have already demonstrated positive usability outcomes; however, these are generally costly and lack necessary EPR integration [36-38]. Applications and EPRs capable of independent integration would offer an ideal solution, leveraging the strengths of both approaches. However, this requires substantial financial resources and close collaboration among the commercial sector, hospitals, health insurance companies, and government.

### Protocol Compliance

A key challenge we encountered was the poor protocol compliance by health care professionals following implementation. During this study inclusion phase, 117/251 (46.6%) of eligible patients were excluded due to improper initiation of the digital pathway, and protocol deviations due to unawareness were high with 14/52 (27%). These rates were unexpectedly high compared to similar studies, highlighting flaws in our implementation process [36,39,40]. An essential area for improvement in this context is the early involvement of diverse health care professionals, particularly those whose daily tasks are altered [31,35,41,42]. This could facilitate integration into daily workflows and consequently improve protocol compliance. This principle also holds true for patients. Existing research in this field underscores the significance of a user-centered design strategy and early stakeholder engagement in the development and implementation of digital tools in daily practice, transforming patients from testers to designers [15,40,43]. Furthermore, using hands-on training and on-the-job guidance for involved health care professionals, complementary to an instructional training system, could yield better results [34,44,45].

### Secondary Health Care Use

Digital treatment pathways effectively reduced secondary health care use by replacing routine function check follow-up appointments with a more patient-centered approach. Notably, the reduction in follow-up appointments exceeded the number of anchor questionnaires sent, suggesting other aspects such as improved patient information and the ability to address concerns via the patient portal and MyChart app also played a role. Our results emphasize the importance of providing patients with an option to schedule an appointment, regardless of questionnaire outcomes as a safety net. Allowing shared decision-making is crucial, as standardized automated systems may not adequately address all concerns and needs [46]. The decline in radiographic imaging likely resulted from fewer routine follow-up appointments, which were previously often linked to routine scheduling of imaging. The added value of follow-up radiographs in extremity injuries has been strongly debated [47]. These findings align with the aims of the digital treatment pathways, in which these radiographs are only performed on clinical indication, rather than routinely. Despite reduced resource use, ED reattendances and complications remained similar, indicating treatment safety was not compromised. These findings are in accordance with current literature; however, future studies with larger sample sizes that focus on the functional outcomes for specific injuries are needed to make definitive statements regarding treatment safety [9].

While the digital pathways improved resource use, their full potential was limited by user-friendliness issues and poor protocol adherence. Addressing these could enhance their benefits. The strain on health care resources and medical personnel warrant reconsideration and innovation of the trauma care system. In this, digitization of the trauma care chain may prove an important tool. However, these new approaches must be carefully designed and (not hastily) implemented, to ensure alignment with the needs of all users.

### Strengths and Limitations

This study has several strengths. First, a concurrent mixed methods design was used, enabling a comprehensive assessment of the digital treatment pathways. Quantitative data measured specific effects, while qualitative data enabled direct interpretation. The concurrent data collection prevented one type of data informing the other, thus ensuring independence of data sources [48,49]. Second, separate teams conducted quantitative and qualitative data collection and analysis independently. Subsequently, they collaboratively engaged in a triangulation process, enhancing the reliability and validity of the results [26]. Additionally, the use of quotes enhanced transparency in presenting qualitative findings [50]. Lastly, using a validated framework provided a structured examination of various feasibility parameters [51].

Several limitations must be addressed. The primary issue is this study’s sample, which consisted of patients with relatively high digital literacy and potentially positive attitudes toward digital tools, due to the convenience sampling method used. This introduces selection bias. Additionally, patients could choose to make an appointment regardless of questionnaire results, meaning those who opted for the digital appointment may have been early adopters. Therefore, our results might not reflect the general patient population accurately, limiting their generalizability, especially to those with lower health literacy or digital skills [44,52]. Future research should validate these findings with a more representative trauma population.

Furthermore, the high protocol noncompliance among health care professionals resulted in a substantial loss of potential study participants. Nonetheless, the final sample size was still deemed sufficient for the purposes of this feasibility study [17,22]. Again, regarding sample representativity, the number of operatively treated patients in our quantitative sample, 30/65 (45%), was relatively high compared to a general monotrauma population. It is unknown whether this influenced our results, this would require a validation study on a larger scale. Importantly, the other baseline characteristics were deemed representative for the monotrauma population at our institution.

Finally, we could not account for the perspective of health care professionals in this study. Future qualitative evaluation in this stakeholder group would be valuable to gain a complete understanding digital treatment pathway feasibility and potentially to identify other points of improvement.

### Conclusion

This study shows that patients with orthopedic trauma consider our digital treatment pathways in follow-up care as feasible and satisfactory, with positive attitudes toward current and future use. This patient-centered approach has the potential to empower patients and reduce secondary health care use. Opportunities to further improve feasibility lie in early stakeholder involvement in design and implementation, integrating specialized tools with existing EPR systems, and providing hands-on training for health care professionals. Clinicians and policy makers can use these insights to better integrate similar digital tools into clinical practice and future guidelines.

## Supplementary material

10.2196/57579 Multimedia Appendix 1 Good Reporting of a Mixed-Methods Study (GRAMMS) checklist.

10.2196/57579 Multimedia Appendix 2 Consolidated Criteria for Reporting Qualitative Studies (COREQ).

10.2196/57579 Multimedia Appendix 3 Full detailed description of the VFC review workflow and the digital treatment pathway workflow. VFC: virtual fracture care.

10.2196/57579 Multimedia Appendix 4 An anonymized version of the patient information letter and consent form.

10.2196/57579 Multimedia Appendix 5 Study surveys at T0, T1, and T2. T: time point.

10.2196/57579 Multimedia Appendix 6 Patient interview topic guide.

10.2196/57579 Multimedia Appendix 7 Quotes per feasibility domain.

## Abbreviations

COREQ: Consolidated Criteria for Reporting Qualitative Studies
ED: emergency department
EPR: electronic patient record
GRAMMS: Good Reporting on a Mixed Methods Study
PROM: patient-reported outcome measure
T0: 1-3 days after emergency department visit
T1: 4-6 weeks after emergency department visit
T2: 10-12 weeks after emergency department visit
