# Supplementary material for: Feasibility of a Web-Based and Mobile-Supported Follow-Up Treatment Pathway for Adult Patients With Orthopedic Trauma in the Netherlands: Concurrent Mixed Methods Study
Source: JMIR Form Res. 2024 Nov 26;8:e57579. doi: 10.2196/57579 (PMC11612530; doi:10.2196/57579)
Supplement: Multimedia Appendix 1 [file formative-v8-e57579-s001.docx]

| **Guideline** | **Page information** |
| --- | --- |
| 1. Describe the justification for using a mixed methods approach to the research question | 6 |
| 2. Describe the design in terms of the purpose, priority and sequence of methods | 6-9 |
| 3. Describe each method in terms of sampling, data collection and analysis | 9-12 |
| 4. Describe where integration has occurred, how it has occurred and who has participated in it | 12 |
| 5. Describe any limitation of one method associated with the present of the other method | 25 |
| 6. Describe any insights gained from mixing or integrating methods | 12-24 |
