# Supplementary material for: Feasibility of a Web-Based and Mobile-Supported Follow-Up Treatment Pathway for Adult Patients With Orthopedic Trauma in the Netherlands: Concurrent Mixed Methods Study
Source: JMIR Form Res. 2024 Nov 26;8:e57579. doi: 10.2196/57579 (PMC11612530; doi:10.2196/57579)
Supplement: Multimedia Appendix 2 [file formative-v8-e57579-s002.docx]

| Domain 1: Research team and reflexivity | | Pages |
| --- | --- | --- |
| Personal Characteristics | | 10-11 |
| 1. Interviewer/facilitator | Which author/s conducted the interview or focus group? K.A.G.J.R. and E.M. *conducted all the interviews.* | 10-11 |
| 2. Credentials | *K.A.G.J.R. PhD; E.M. PhD;.* | 10-11 |
| 3. Occupation | *K.A.G.J.R. - Assistant Professor; E.M. Thinc. researcher;* | N.a. |
| 4. Gender | *Female* | N.a. |
| 5. Experience and training | *Extensive training and over 6 years of experience in qualitative research.* | N.a. |
| Relationship with participants | |  |
| 6. Relationship established | Was a relationship established prior to study commencement? *No* | 11 |
| 7. Participant knowledge of the interviewer | What did the participants know about the researcher? *The researcher introduced herself at the start of the interviews as a researcher with extensive experience in qualitative research.* | N.a. |
| 8. Interviewer characteristics | What characteristics were reported about the interviewer/facilitator? e.g., Bias, assumptions, reasons, and interest in the research topic?  *K.A.G.J.R. - “An academic social scientist.” E.M. “an expert in nursing research.”* | 10-11 |
| Domain 2: study design | |  |
| Theoretical framework | |  |
| 9. Methodological orientation and Theory | What methodological orientation was stated to underpin the study? e.g., grounded theory, discourse analysis, ethnography, phenomenology, content analysis?  *A thematical analysis was performed [2, 3].* | 11-12 |
| Participant selection | |  |
| 10. Sampling | How were participants selected? e.g., purposive, convenience, consecutive, snowball?  *Patients were recruited using a convenience sampling method. Patients who presented at the ED for treatment of a musculoskeletal injury were encouraged to activate an account for the online patient portal.* | 9 |
| 11. Method of approach | How were participants approached? e.g., face-to-face, telephone, mail, email?  *Patients were informed about the study by a researcher the day after their ED visit via an information e-mail and by phone and were sent an informed consent form if they indicated willingness to participate.* | 9 |
| 12. Sample size | How many participants were in the study?  *Fifteen interviews were conducted.* | 12-13 |
| 13. non-participation | How many people refused to participate or dropped out? Reasons?  *Of those who showed interested in the study, four dropped out. No reasons were obtained.* | 12-13 |
| Setting | |  |
| 14. Setting of data collection | Where was the data collected? e.g., home, clinic, workplace?  *All interviews were conducted using Microsoft Teams.* | 10 |
| 15. Presence of non-participants | Was anyone else present besides the participants and researchers?  *No.* | N.a. |
| 16. Description of sample | What are the important characteristics of the sample? e.g., demographic data, date?  *Of the 66 patients included for the quantitative part of the study, 30 (45%) patients consented to interview participation. Seventeen (26%) participants were scheduled for an interview and fifteen (23%) patients eventually participated in the interviews (Table 4). The reasons for not participating could not be obtained. Nine (60%) of interviewed patients were female and six (40%) were male. Age ranged between 23 and 77 years, with a mean age of 48.* | 11-12 |
| Data collection | |  |
| 17. Interview guide | Were questions, prompts, guides provided by the authors? Was it pilot tested?  *Semi-structured in-depth interviews were conducted using an interview topic guide.* | 10-11 |
| 18. Repeat interviews | Were repeat interviews carried out? If yes, how many?  *No.* | N.a. |
| 19. Audio/visual recording | Did the research use audio or visual recording to collect the data?  *All interviews were audio recorded.* | 11 |
| 20. Field notes | Were field notes made during and/or after the interview or focus group?  *Notes were taken during the interviews to describe nonverbal communication.* | 11 |
| 21. Duration | What was the duration of the interviews or focus group?  *45 to 90 minutes.* | 10 |
| 22. Data saturation | Was data saturation discussed?  *The sampling was scheduled to stop based on the principle of data saturation and exactly determined a posteriori.[4]* | 9 |
| 23. Transcripts returned | Were transcripts returned to participants for comment and/or correction?  *No.* | N.a. |
| Domain 3: analysis and findings | |  |
| Data analysis | |  |
| 24. Number of data coders | How many data coders coded the data?  *Two researchers* | 11 |
| 25. Description of the coding tree | Did authors provide a description of the coding tree?  *No.* | N.a. |
| 26. Derivation of themes | Were themes identified in advance or derived from the data?  *No themes were identified in advance.* | N.a. |
| 27. Software | What software, if applicable, was used to manage the data?  *NVivo version 20.[5]* | 11 |
| 28. Participant checking | Did participants provide feedback on the findings?  *No.* | N.a. |
| Reporting | |  |
| 29. Quotations presented | Were participant quotations presented to illustrate the themes / findings? Was each quotation identified? e.g., participant number?  *Yes.* | 13-20 / Table 3 |
| 30. Data and findings consistent | Was there consistency between the data presented and the findings?  *Yes.* | N.a. |
| 31. Clarity of major themes | Were major themes clearly presented in the findings?  *Yes.* | 13-20 |
| 32. Clarity of minor themes | Is there a description of diverse cases or discussion of minor themes?  *Themes and sub-themes are discussed in the results.* | 13-20 |
