# Supplementary material for: Feasibility of a Web-Based and Mobile-Supported Follow-Up Treatment Pathway for Adult Patients With Orthopedic Trauma in the Netherlands: Concurrent Mixed Methods Study
Source: JMIR Form Res. 2024 Nov 26;8:e57579. doi: 10.2196/57579 (PMC11612530; doi:10.2196/57579)
Supplement: Multimedia Appendix 3 [file formative-v8-e57579-s003.docx]

*VFC review protocol*

At our institution, orthopedic trauma patients who required follow-up treatment at the hospital were managed according to a VFC review protocol. With this protocol, ED healthcare professionals electronically referred patients to a multidisciplinary VFC review meeting scheduled for the next workday, during which comprehensive treatment plans were assigned to each referred patient. These plans included all required follow-up appointments (telephone or face-to-face) and imaging, with a final routine follow-up appointment at 8-10 weeks generally included to check on functional recovery. Patients were informed by phone of their treatment options directly after VFC review and consent for definitive treatment was discussed. After reaching consent, the complete follow-up treatment was scheduled and documented in the EPR.

*Digital treatment pathways*

In addition to the VFC review treatment plans, supplementary digital treatment pathways were introduced on October 1^st^ of 2022 for the eight most frequently seen types of hand, wrist, ankle and foot fractures, including both non-operative and operative treatment pathways (Table 1). The mode of delivery for the digital treatment pathways was the online patient portal: and its accessory app, both an extension of the EPR system: Epic. To gain access, patients were required to activate a personal account. After physical identification with an ID-card or passport in the ED by an administrative assistant, patients received an activation code by e-mail, which could be used to activate full access to their personal account. To promote the use of the digital treatment pathways, orthopedic trauma patients who presented at the ED were specifically encouraged by the administrative assistants to activate their account directly after the ED visit.

The digital treatment pathways had several functionalities. They provided patients with additional information, including digital leaflets and animated instructional videos covering various aspects (e.g. injury-related information, cast/brace related information, exercise regimes). Furthermore, patient recovery was monitored remotely using specific PROMs questionnaires, selected based on consensus among orthopedic surgical staff (Table 1). Criteria for selection included availability in the online patient portal and app, question count (to minimize patient effort), and staff's clinical experience with PROMs scores. In addition to PROMs questionnaires, several digital treatment pathways included an additional anchor questionnaire prior to the final routine function check appointment at 8-10 weeks. Patients’ answers to the first and third question of this questionnaire determined the need for this appointment (by phone or face-to-face) (Figure S1).


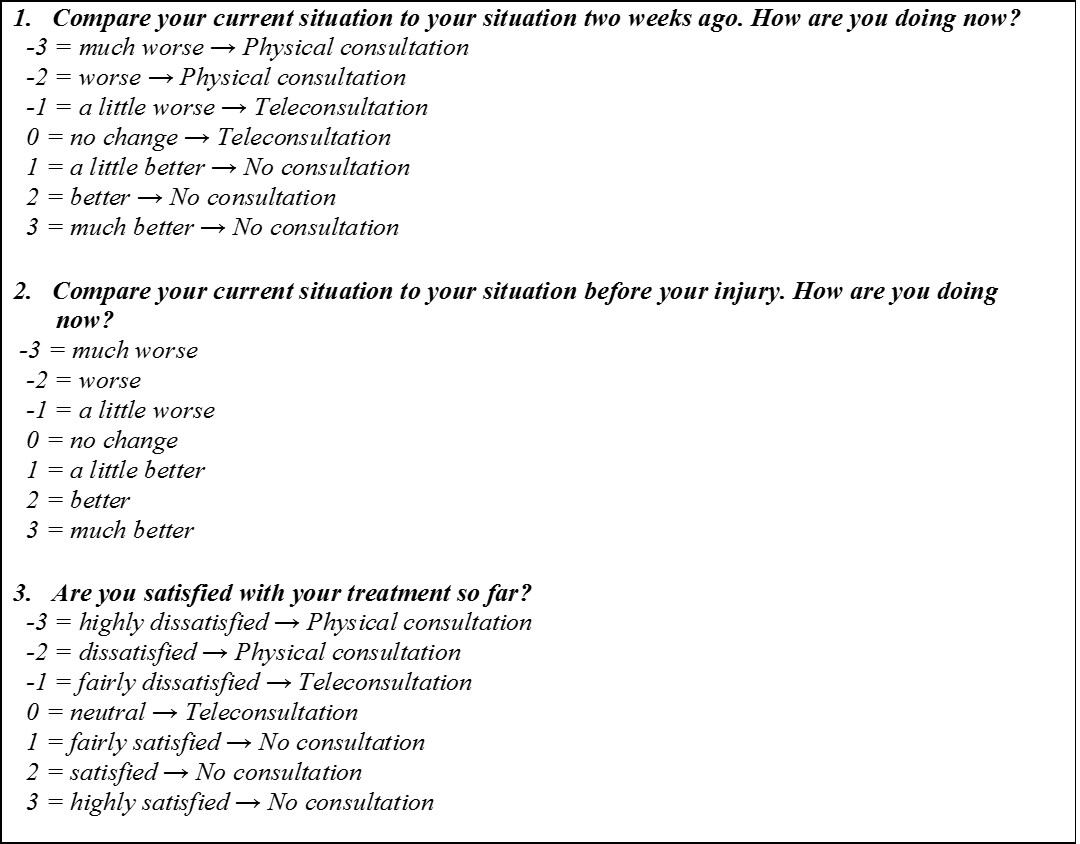
**Figure S1.** The anchor questionnaire and linked results

This anchor questionnaire and its consequences were developed, discussed and adjusted by orthopedic surgical staff until consensus was reached. After questionnaire completion, patients received immediate automated feedback on whether a follow-up appointment was indicated and in which form (face-to-face or by phone) via the online patient portal and the accessory app. Results were also transmitted to outpatient clinic administrative staff via the EPR, who scheduled appointments accordingly within two days. Non-responding patients received reminders after three and five days via messages in their online patient portal and via push-notifications in the app. As a safety net, patients who did not complete the anchor questionnaires despite these reminders were scheduled for a routine follow-up appointment by phone. Furthermore, patients could also request a follow-up appointment regardless of questionnaire outcomes. The digital pathways also enabled patients to directly ask their treatment team questions, which were addressed within 24 hours by an outpatient clinic assistant.

The digital treatment pathways were launched electronically by healthcare professionals directly after the VFC review meeting. This process was facilitated through a pop-up window integrated into the current Epic navigator menu, requiring one extra mouse click by the healthcare professional. Once initiated, the pathways ran automatically and healthcare professionals could access questionnaire results within the existing Epic navigator menu prior to follow-up appointments. To familiarize healthcare professionals with the new pathways, they received instructions from the VFC project team and supporting staff of the online patient portal through three presentations, three informative emails, and a readily accessible tip sheet in the hospital-wide protocol file system.
