# Supplementary material for: Feasibility of a Web-Based and Mobile-Supported Follow-Up Treatment Pathway for Adult Patients With Orthopedic Trauma in the Netherlands: Concurrent Mixed Methods Study
Source: JMIR Form Res. 2024 Nov 26;8:e57579. doi: 10.2196/57579 (PMC11612530; doi:10.2196/57579)
Supplement: Multimedia Appendix 4 [file formative-v8-e57579-s004.docx]

**Virtual Fracture Care – Digital treatment pathways**

**Patient information letter, Version 1.0**

Dear patient,

In our hospital, we are not only involved in the treatment of patients, but also strive to improve future care through conducting scientific research. Through this letter, we ask for your participation in one of these medical-scientific studies. Participation is voluntary. You decide whether to participate. Your choice does not affect the care you receive. In this information letter, you will learn more about the study.

1. **General information**

The study is conducted by researchers from the study institution and an external research agency. You are asked to participate because you are being treated at the study institution for a fracture or other injury and are using digital support for your treatment.

**2. What is the purpose of the study?**

For the treatment of your fracture or other injury, a treatment plan has been made. This treatment plan is called Virtual Fracture Care and it is offered digitally through the online patient portal and/or the associated app. This allows healthcare providers to monitor your health and recovery remotely, and a follow-up appointment is not always necessary. The aim of the study is to investigate whether Virtual Fracture Care works well and what users think of it.

1. **Wat does participation mean for you?**

This study consists of 2 parts:

Part 1 of the study (questionnaires)

For the study, we ask you to complete a questionnaire three times. The questions are about your treatment with Virtual Fracture Care. You can fill out the questionnaires via the online patient portal and/or the associated app, or they will be sent to your email address. Filling out each questionnaire takes a maximum of 8 minutes. The questionnaires will be sent to you in the first week of your treatment and after 4 and 12 weeks. For the study, data will be extracted from your medical record. This includes your gender, age, and type of injury. We also keep track of how often you use the patient portal and/or the associated app. During the study, we may ask you to participate in an online interview. This is optional. You are entirely free to say 'no' to this.

Part 2 of the study (Online interview)

If you indicate that you may be approached for this, you may be asked to participate in an interview about your experience with the treatment. The interview will be conducted 8-12 weeks after your hospital visit using video calling with one of the researchers. During the interview, an audio recording will be made. With the help of the audio recording, the researchers can transcribe the interview afterward. The audio recording will not be shared with anyone and will be deleted after transcription. The interview is voluntary. You can also only participate in part 1 of the study (questionnaires). You can indicate your choice on the consent form.

**4. When does the study end?**

The study ends after you have completed the third questionnaire (and potentially completed the interview). If you participate, you can always stop during the study. You do not need to provide a reason for this. Your data collected up to that point will still be used and stored for the study. More information about this is available in appendix 1.

**5. What do we do with your data?**

If you participate, you give consent for your data to be collected, used, and stored. It concerns the following data:

- Your name;
- Your email address;
- The questionnaires you filled out;
- Your gender;
- Your age;
- What type of complex injury you have;

We comply with the General Data Protection Regulation. You can read more about this in attachment 1. If you indicate that you want to participate in an online interview, your contact details will be shared with the relevant members of research team. These are only available to the researchers directly involved in this study.

**6. Do you have any questions?**

You can ask the researchers any questions about the study. You can find the contact details at the bottom of this information letter.

***Hoe te handelen bij klachten?***

*If you have any complaints about the study, you can report this to the researcher. If you prefer not to do this, you can contact the complaints officer of the study institution.*

On behalf of all researchers, Thank you for your time.

Attachments:

1. Additional information about the processing of your data
2. Consent form

**Attachment 1: Additional information about the processing of your data**

**How do we protect your privacy?**

To protect your privacy, we assign a code to your data. We keep the key to the code in a secure location in the study institution. When we process your data, we only use this code. In reports and publications about the study, no one can trace this code to you.

**Who will be able to see your data?**

Some individuals can see your name and other personal data. These are people who ensure that the researchers conduct the study properly and reliably. These individuals keep your data confidential. We ask for your consent for this. These individuals are:

- National authorities such as the Health and Youth Care Inspectorate.

**Hoelang en waar bewaren wij uw gegevens?**

We store your data on a secure drive at the study institution en het UMC Utrecht.

**Can you withdraw your consent for the use of your data?**

You can decide at any time not to participate or to stop. You do not need to provide a reason. If you withdraw your consent for the use of your data and the researchers have already collected data from you, they may still use and store this data.

**Do you want to know more about your privacy?**

- Do you want to know more about your rights regarding the processing of your data? Then visit [www.autoriteitpersoonsgegevens.nl](http://www.autoriteitpersoonsgegevens.nl/).
- If you have complaints about the processing of your data, we recommend that you discuss this first with the researchers. You can also file a complaint with the Dutch Data Protection Authority via [https://www.autoriteitpersoonsgegevens.nl](https://www.autoriteitpersoonsgegevens.nl/).

**Attachment 2: Consent form for study participation**

**Virtual Fracture Care – digital treatment pathways**

• I have read the information letter. I was also able to ask questions. My questions have been adequately answered. I had enough time to decide whether to participate.

• I understand that participation is voluntary. I also know that I can decide at any time not to participate or to stop. I do not need to provide a reason for this.

• I consent to the collection, use, and storage of my data.

• I understand that for the monitoring of the study, some individuals may have access to my name and other personal data. These individuals are listed in attachment 1. I give consent for this.

• I want to participate in the following parts (Check which ones apply to you):

- Part 1 (questionnaires): ☐
- Part 2 (online interview): ☐

Participant's Name: ……………………………………

Signature: …………………………………… Date:

I declare that I have fully informed this participant about the study.

Researcher's Name (or representative): ……………………………………

Signature: …………………………………… Date:
