# Supplementary material for: Feasibility of a Web-Based and Mobile-Supported Follow-Up Treatment Pathway for Adult Patients With Orthopedic Trauma in the Netherlands: Concurrent Mixed Methods Study
Source: JMIR Form Res. 2024 Nov 26;8:e57579. doi: 10.2196/57579 (PMC11612530; doi:10.2196/57579)
Supplement: Multimedia Appendix 5 [file formative-v8-e57579-s005.docx]

**Survey T0**

For the treatment of your fracture or other injury, a digital treatment pathway has been created, which is offered through The patient portal and/or the accessory app. The following questions are about your expectations regarding the use of the patient portal and/or the accessory app.

1. I expect that I am able to use the patient portal and/or the accessory app with the explanations and instructions I have received.

- Completely disagree
- Disagree
- Agree
- Completely agree

2. I expect that using the patient portal and/or the accessory app will assist me in my treatment and recovery.

- Completely disagree
- Disagree
- Agree
- Completely agree

Do you expect that using the patient portal and/or the accessory app will have consequences for communication with your treatment team?

- Yes
- No

3a. Can you further explain your answer?

*...*

**Survey T1**

For the treatment of your fracture or other injury, a digital treatment pathway has been created, which is offered through the patient portal and/or the accessory app. The following questions are about your experiences with the use of the patient portal and/or the accessory app.

1. How satisfied are you with the use of the patient portal and/or the accessory app? Rate on a scale from 0 to 10 (0 = very dissatisfied and 10 = very satisfied).

0 – 1 – 2 – 3 – 4 – 5 – 6 – 7 – 8 – 9 – 10

1a. What is the main reason for this rating?

...

2. I am able to use the patient portal and/or the accessory app with the explanations and instructions I have received.

- Completely disagree
- Disagree
- Agree
- Completely agree

3. The use of the patient portal and/or the accessory app helps me in my treatment and recovery.

- Completely disagree
- Disagree
- Agree
- Completely agree

4. I feel secure in using the patient portal and/or the accessory app.

- Completely disagree
- Disagree
- Agree
- Completely agree

5. The amount of messages I receive through the patient portal and/or the accessory app is sufficient.

- Completely disagree
- Disagree
- Agree
- Completely agree

6. The use of the patient portal and/or the accessory app meets my expectations.

- Completely disagree
- Disagree
- Agree
- Completely agree

7. How satisfied are you with the communication with your treatment team? Rate on a scale from 0 to 10 (0 = very dissatisfied and 10 = very satisfied).

0 – 1 – 2 – 3 – 4 – 5 – 6 – 7 – 8 – 9 – 10

7a. What is the main reason for this rating?

...

8. How likely is it that you would use the patient portal and/or the accessory app again? Rate on a scale from 0 to 10 (0 = very unlikely and 10 = very likely).

0 – 1 – 2 – 3 – 4 – 5 – 6 – 7 – 8 – 9 – 10

8a. What is the main reason for this rating?

...

9. How likely is it that you would recommend the use of the patient portal and/or the accessory app to others? Rate on a scale from 0 to 10 (0 = very unlikely and 10 = very likely).

0 – 1 – 2 – 3 – 4 – 5 – 6 – 7 – 8 – 9 – 10

9a. What is the main reason for this rating?

...

**Survey T2**

For the treatment of your fracture or other injury, a digital treatment pathway has been created, which is offered through the patient portal and/or the accessory app. The following questions are about your experiences with the use of the patient portal and/or the accessory app.

1. How satisfied are you overall with your treatment? Rate on a scale from 0 to 10 (0 = very dissatisfied and 10 = very satisfied).

0 – 1 – 2 – 3 – 4 – 5 – 6 – 7 – 8 – 9 – 10

1a. What is the main reason for this rating?

...

2. How satisfied are you overall with the information provision during your treatment? Rate on a scale from 0 to 10 (0 = very dissatisfied and 10 = very satisfied).

0 – 1 – 2 – 3 – 4 – 5 – 6 – 7 – 8 – 9 – 10

2a. What is the main reason for this rating?

...

3. How satisfied are you with the communication with your treatment team? Rate on a scale from 0 to 10 (0 = very dissatisfied and 10 = very satisfied).

0 – 1 – 2 – 3 – 4 – 5 – 6 – 7 – 8 – 9 – 10

3a. What is the main reason for this rating?

...

4. How satisfied are you with the use of the patient portal and/or the accessory app? Rate on a scale from 0 to 10 (0 = very dissatisfied and 10 = very satisfied).

0 – 1 – 2 – 3 – 4 – 5 – 6 – 7 – 8 – 9 – 10

4a. What is the main reason for this rating?

...

5. I was able to use the patient portal and/or the accessory app with the explanations and instructions I have received.

- Completely disagree
- Disagree
- Agree
- Completely agree

6. The use of the patient portal and/or the accessory app helped me in my treatment and recovery.

- Completely disagree
- Disagree
- Agree
- Completely agree

7. I felt secure in using the patient portal and/or the accessory app.

- Completely disagree
- Disagree
- Agree
- Completely agree

8. The amount of messages I received through the patient portal and/or the accessory app was sufficient.

- Completely disagree
- Disagree
- Agree
- Completely agree

9. The use of the patient portal and/or the accessory app met my expectations.

- Completely disagree
- Disagree
- Agree
- Completely agree

10. How likely is it that you would use the patient portal and/or the accessory app again? Rate on a scale from 0 to 10 (0 = very unlikely and 10 = very likely).

0 – 1 – 2 – 3 – 4 – 5 – 6 – 7 – 8 – 9 – 10

10a. What is the main reason for this rating?

...

11. How likely is it that you would recommend the use of the patient portal and/or the accessory app to others? Rate on a scale from 0 to 10 (0 = very unlikely and 10 = very likely).

0 – 1 – 2 – 3 – 4 – 5 – 6 – 7 – 8 – 9 – 10

11a. What is the main reason for this rating?

...
