# Supplementary material for: Feasibility of a Web-Based and Mobile-Supported Follow-Up Treatment Pathway for Adult Patients With Orthopedic Trauma in the Netherlands: Concurrent Mixed Methods Study
Source: JMIR Form Res. 2024 Nov 26;8:e57579. doi: 10.2196/57579 (PMC11612530; doi:10.2196/57579)
Supplement: Multimedia Appendix 6 [file formative-v8-e57579-s006.docx]

| Interview topic guide | |
| --- | --- |
| Topic | **Example question or description** |
| Introduction interviews | Interviewer introduced the interview |
| Acceptability | “Can you share your experience with “*The patient portal and/or the accessory app”*?”  “Can you elaborate on your satisfaction about “*the patient portal and/or the accessory app”*?”  “What were your expectations about “*the patient portal and/or the accessory app”* before using the app?” |
| Demand | “How often did you use *“The patient portal and/or the accessory app”*?”  “How likely is it that you would use *“The patient portal and/or the accessory app”* again?”  “How likely is it that you would recommend using *“The patient portal and/or the accessory app”* to others?” |
| Implementation | “How would you describe using *“The patient portal and/or the accessory app”*?”  “Who can use *“The patient portal and/or the accessory app”* according to you?”  “What are factors that make it difficult to use *“The patient portal and/or the accessory app”*?”  “What are factors that make it easy to use *“The patient portal and/or the accessory app”?”* |
| Integration | “How was the use of *“The patient portal and/or the accessory app”* in your daily life?” |
| Preliminary efficacy | “How was the follow-up in your care?” |
| Value | “How would you summarize your experience with *“The patient portal and/or the accessory app”*? |
| Involvement | “When do you feel involved with the care you receive?”  “In what way did *“The patient portal and/or the accessory app”* contribute to you feeling involved with your treatment?” |
| Closing | Closing of the interview. |
