# Supplementary material for: Feasibility of a Web-Based and Mobile-Supported Follow-Up Treatment Pathway for Adult Patients With Orthopedic Trauma in the Netherlands: Concurrent Mixed Methods Study
Source: JMIR Form Res. 2024 Nov 26;8:e57579. doi: 10.2196/57579 (PMC11612530; doi:10.2196/57579)
Supplement: Multimedia Appendix 7 [file formative-v8-e57579-s007.pdf]

Theme	#	Participant	Quote
**Acceptability**			
Expectations	#1	P12. Female, 59 years old	"When you say '*there's a treatment plan in that app*' I'm thinking: so… what is the treatment plan in that app? ...it's actually mainly for appointments. I don't really see other things in there. … I wanted to see what the results of examinations were and things like that. … Well, I'm really not stupid but I just don't get to see those. "
Experiences	#2	P33. Female, 76 years old	"I received a questionnaire to fill out – what I could and couldn't do, what was painful, what wasn't painful. And then I immediately received a message that at that moment, it wasn't necessary to come in for an appointment. And I found that nice, that it could be done online instead of having to go to the hospital just to answer those questions. And a few weeks later, I reviewed that questionnaire, which I had filled out at the time. And I compared the answers with how things were at that moment. And then I thought, 'Oh, I couldn't do that then, and now I can.' So I found that was nice. "
	#3	P35. Male, 72 years old,	"Well in the end of course it's about the result, right. I think they took great care of me, and it led to a great recovery. That is of course very important. Second, I found the people very friendly and very professional, that is both the ward and later also in the surgery. Well, I found that went very well and very pleasant. Thirdly, the use of the app. I just found that very pleasant - to use that tool with it, to make that process go smoothly."
Satisfaction	#4	P63. Female, 26 years old	Interviewer: "On a scale of one to ten, what grade would you give the app?"<br>Participant: "A seven, I think. A definite pass."<br>Interviewer: "What would we have to do to turn that seven into an eight or even a nine?"<br>Participant: "I think just making it a little clearer where everything is. So that you are not searching: *'oh, where can I find that one thing again?'*
**Demand**			
Actual use	#5	P12. Female, 59 years old	"I think I just got some [notification of *"MijnOLVG"*] again, so I can do it. Where did I get it? At least I thought I saw a notification come in *"MijnOLVG"*. Oh yes! I then choose to log in with Face ID because the other option is DigiD. Well, then I can choose: a code via text message or via e-mail. Well, I then choose by text message. It pops up: 018461. So that way it's pretty simple and then... Yes, so now in *"MijnOLVG"* looking at it like, 'what came in?' [notification] Let's see. Then first I see it says: appointment scheduled: January 25. Well, that was this morning, I've been there already. And I'm thinking: 'Where's the notification from?' … Oh, wait a minute. Now I see it says x-ray, but that's for Friday, so that's an upcoming appointment. And under [this upcoming appointment] I now see it says visit summary. From my conversation with the surgeon this morning ... Then it says visit today: 'You saw Mrs. Do on Wednesday, January 25.' That's it. So that's not what I imagined from a visit summary. ... And again, I'm thinking, ' am I missing something?' You start doubting yourself again."
Intended use	#6	P33. Female, 76 years old	"I think I would [use it again] yes… *Interviewer: would you recommend other people to use it?* I hope it's not necessary, but yes absolutely. … I would just say use all the options [in the *MijnOLVG* application]. You can find everything there."

**Implementation**			
	#7	P35. Male, 72 years old	"And such a tool helps to communicate easily. Those are the three factors that together give a good feeling... I also find it something of this time. Modern, and that the hospital is adapting to the use of tools of this time. It also expresses something about the focus: 'we are also trying to use a digital instrument to facilitate the entire process."
**Integration**			
	#8	P29. Female, 54 years old	"No, because it is very linguistic oriented. I think if you're a little bit less [literate] and a little bit older, then you may experience a bit of trouble with that. And yes, it [*MijnOLVG*] is something for people who are handy with phones and with iPads. It's also very linguistically oriented, very few pictures in the app."
**Limited efficacy**			
	#9	P50. Female, 29 years old	"So, then I went to the hospital to have the cast removed, and actually, after that, I haven't had any human contact with anyone from the hospital anymore, only through that app with the questionnaire. ... I have to say, I found that quite positive in itself because things were going well at that point, so it also feels a bit pointless and a waste of everyone's time to have another conversation about it. "
	#10	P69. Male, 40 years old	"No, I thought that was very strange [that no follow-up appointment was scheduled after continued pain and mobility issues]. I mean I know what I feel. I assume that the app [*MijnOLVG*] has certain parameters by which it determines if it is okay or if you need to have an appointment. Apparently how I entered it fell under the threshold of "no appointment needed". But it is very difficult to have a computer determine that. Pain and functionality symptoms are relative and yet very subjective."
